# Measuring maladaptive avoidance: from animal models to clinical anxiety

**DOI:** 10.1038/s41386-021-01263-4

**Published:** 2022-01-15

**Authors:** Tali M. Ball, Lisa A. Gunaydin

**Affiliations:** 1 Department of Psychiatry and Behavioral Sciences, Stanford University School of Medicine, Stanford, CA 94305 USA; 2 Department of Psychiatry and Behavioral Sciences, University of California San Francisco, San Francisco, CA 94143 USA; 3 Kavli Institute for Fundamental Neuroscience, University of California San Francisco, San Francisco, CA 94143 USA

**Keywords:** Emotion, Anxiety

## Abstract

Avoiding stimuli that predict danger is required for survival. However, avoidance can become maladaptive in individuals who overestimate threat and thus avoid safe situations as well as dangerous ones. Excessive avoidance is a core feature of anxiety disorders, post-traumatic stress disorder (PTSD), and obsessive-compulsive disorder (OCD). This avoidance prevents patients from confronting maladaptive threat beliefs, thereby maintaining disordered anxiety. Avoidance is associated with high levels of psychosocial impairment yet is poorly understood at a mechanistic level. Many objective laboratory assessments of avoidance measure *adaptive* avoidance, in which an individual learns to successfully avoid a truly noxious stimulus. However, anxiety disorders are characterized by *maladaptive* avoidance, for which there are fewer objective laboratory measures. We posit that maladaptive avoidance behavior depends on a combination of three altered neurobehavioral processes: (1) threat appraisal, (2) habitual avoidance, and (3) trait avoidance tendency. This heterogeneity in underlying processes presents challenges to the objective measurement of maladaptive avoidance behavior. Here we first review existing paradigms for measuring avoidance behavior and its underlying neural mechanisms in both human and animal models, and identify how existing paradigms relate to these neurobehavioral processes. We then propose a new framework to improve the translational understanding of maladaptive avoidance behavior by adapting paradigms to better differentiate underlying processes and mechanisms and applying these paradigms in clinical populations across diagnoses with the goal of developing novel interventions to engage specific identified neurobehavioral targets.

## Introduction

Avoidance is a central and highly impairing feature of anxiety and related disorders, preventing individuals from engaging fully in their lives [1]. Avoidance refers to any behavior that allows an individual to minimize exposure to stimuli or situations that are unpleasant, distressing, or threatening. While avoiding stimuli that predict danger is required for survival, avoidance can become maladaptive when an individual avoids situations that are relatively safe, resulting in negative consequences [2]. This maladaptive avoidance is a core feature of anxiety disorders and related conditions such as post-traumatic stress disorder (PTSD) and obsessive-compulsive disorder (OCD) [3]. For example, avoidance is a diagnostic feature of social anxiety disorder and PTSD, and excessive worry in generalized anxiety disorder may allow patients to avoid distressing visual imagery, negative emotional contrast, or feeling unsafe [4, 5].

Avoidance is a major driver that maintains anxiety pathology; it is an appealing coping strategy because it effectively reduces anxiety in the short term, but can maintain anxiety in the long term by preventing patients from learning that their feared situations are, in fact, safe [6]. Reducing avoidance is, therefore, an important treatment goal and a crucial part of interventions such as exposure therapy (e.g., [7]). However, while we have a thorough translational understanding of the neural mechanisms underlying defensive reactions to acute threats such as fear and freezing, mechanisms underlying avoidance are comparatively unexplored, representing a major gap in our understanding of anxiety pathology [8].

To advance our understanding of avoidance behavior and its neural mechanisms, it will be critical to objectively measure avoidance [9]. Current clinical assessment of avoidance behavior largely relies on retrospective self-report (though novel approaches based on passive smartphone sensing are under development [10]). Due to the often subtle and ingrained nature of avoidance, however, retrospective self-report may not be reliable. Furthermore, most clinical assessment of avoidance is disorder-specific (e.g., [11]), and assessment of avoidance independent of excessive fear is lacking. There is, therefore, an unmet need for objective behavioral measurement of avoidance, outside the context of any one disorder. Such measurement would facilitate comparison across individuals, across anxiety pathology, and across species, making it critical for mechanistic laboratory work as well as trans-diagnostic application.

In this paper, we will present three biobehavioral processes that may underlie maladaptive avoidance, describe how avoidance is most commonly measured in laboratory settings in both animals and humans, and summarize what is already known about its neural mechanisms. We then discuss how existing paradigms could be modified to improve our understanding of avoidance behavior and identify more precise novel targets for tailored intervention. This narrative review therefore complements and extends existing reviews of avoidance mechanisms in rodents [12], avoidance learning in clinical anxiety [2, 13, 14], anticipation and uncertainty [15], and approach-avoidance conflict [16].

## A neurobehavioral model of maladaptive avoidance behavior

A key translational need is to understand *maladaptive* avoidance, which we define as avoidance of a relatively safe stimulus often resulting in negative consequences for the individual. Existing laboratory paradigms to study maladaptive avoidance have, therefore, examined avoidance in relative safety or in situations where negative consequences follow from the avoidance behavior. However, even within each of these laboratory paradigms, observed avoidance behavior could be driven by different underlying processes. Throughout this review, we will discuss three neurobehavioral processes that could drive maladaptive avoidance: (i) heightened threat appraisal, (ii) habitual avoidance, and (iii) trait avoidance tendency, defined below. Note that while there is a technical distinction between the terms ‘fear’ and ‘anxiety’ related to acuity of threat, both acute and distant threats can lead to avoidance behavior [17], and we therefore discuss studies of both fear and anxiety.

By **heightened threat appraisal**, we mean a tendency to overestimate threat, leading to a higher level of fear evoked by a stimulus. Heightened threat appraisal can, therefore, drive excessive avoidance behavior because of the relationship between fear and avoidance [18]. When heightened threat appraisal is primarily responsible for maladaptive avoidance, the cause of the problem may therefore be upstream of the avoidance behavior itself, and the avoidance may simply be a byproduct of heightened fear. For example, individuals with panic disorder may erroneously perceive an elevated heart rate from exercise as dangerous, and subsequently avoid exercising. In this case, the mistaken perception of elevated heart rate as dangerous is the issue, and in a situation where elevated heart rate was indeed dangerous, the same avoidance behavior would not be considered maladaptive. This neurobehavioral model is consistent with Mowrer’s classical two-factor theory, which proposes that avoidance is primarily motivated by high levels of fear and reinforced by fear reduction [19, 20]. However, more contemporary work has demonstrated that fear and avoidance may not always be so tightly coupled (as reviewed in [14]), and avoidance may be driven by other processes independent of high fear levels, such as habitual avoidance and/or trait avoidance tendency.

**Habitual avoidance** may emerge when avoidance is repeatedly reinforced over time. Avoidance habits are cue-based, non-goal-directed behaviors that are insensitive to outcomes [21], and have been particularly implicated in OCD as one way to conceptualize compulsive behavior [22]. For example, a behavior such as excessive lock-checking may initially be driven by the goal of preventing burglary, then reinforced by the feeling of brief relief from anxiety, and eventually become a habitual response to the cue of leaving home. In this case, the habitual avoidance behavior may no longer be goal directed, and unlike avoidance driven by threat appraisal, habitual avoidance can become decoupled from the level of fear an individual feels.

By **trait avoidance tendency**, we mean that there may be stable individual differences such that even when experiencing comparable threat appraisal, some individuals may have an innate propensity to avoid to a greater extent. As with habitual avoidance, trait avoidance tendency would therefore likely be decoupled from the level of fear (as in [23]). However, in contrast to habitual avoidance, trait avoidance tendency implies high levels of avoidance broadly across situations, rather than specific to particular reinforced or over-learned behaviors. Although there are effective interventions that address threat appraisals (e.g., cognitive therapy [24]) and habitual behavior (e.g., habit reversal [25]), assessment and treatment of broad trait avoidance tendencies remain relatively underdeveloped.

## Adaptive avoidance

As a foundation to understanding maladaptive avoidance, we first review existing paradigms that assess adaptive avoidance, in which individuals avoid a truly noxious stimulus. Adaptive avoidance behaviors fall into two broad categories: “active avoidance”, which is defined as performing a learned action to prevent harm; and “passive avoidance”, which is defined as withholding a behavioral response to prevent harm [12]. In rodents, active avoidance has been traditionally studied using a “shuttle box” consisting of two chambers with metal bar floors capable of delivering shocks. A neutral sensory cue (the conditioned stimulus, or CS; e.g., an auditory tone) is presented in one of the chambers and signals an impending electric shock (the unconditioned stimulus, or US). Animals learn that “shuttling” to the other chamber in response to the CS prevents the shock from occurring. Active avoidance is thought to involve two types of learning: (i) Pavlovian fear conditioning, in which animals learn the CS-US association (i.e., the sensory cue predicts shock); and (ii) instrumental conditioning, in which animals learn that shuttling prevents shock [26, 27]. During early active avoidance training, Pavlovian processes dominate, as animals exhibit fearful freezing responses. Gradually throughout training, instrumental processes dominate as animals learn that they can avoid the shock by shuttling. This paradigm is considered a measure of adaptive avoidance because animals are avoiding the very real threat of an electric shock. Variations include “platform-mediated avoidance” [28], which adds a “safe” platform in one corner where the animals can step to avoid the shock, and Sidman avoidance [29, 30], in which an aversive outcome occurs at a regular time interval instead of in response to a cue, resulting in repeated uncued avoidance behavior. A related type of task, called aversive Pavlovian Instrumental Transfer (PIT), explicitly separates the Pavlovian and instrumental learning processes enabling investigations into the relationship between each of these learning processes [31, 32].

The active avoidance paradigm has also been adapted for use in human research with three major differences. First, human analogues typically do not involve moving to a different location to avoid an aversive outcome but often rely on behaviors such as a button press in computer-based tasks (though see also [33]). Second, in human analogues the experimenter often verbally instructs the participant on the CS-US association, resulting in a minimal contribution of Pavlovian learning. When Pavlovian learning is included in the paradigm, it usually is conducted without any opportunity to engage in avoidance behavior [34]. This has the benefit of isolating the fear and avoidance learning components, with the drawback of fewer parallels to rodent paradigms. Third, while in rodent paradigms avoidance behavior is always learned through trial-and-error, human avoidance paradigms can either be learned (e.g., “one of these buttons will prevent shock”; [35]) or fully instructed (e.g., “press here to prevent shock”; [36]).

Rodent and human studies employing these active avoidance tasks have converged on three critical neural structures: the medial prefrontal cortex (mPFC), the amygdala, and the striatum [28, 30, 35, 37–40]. The mPFC is implicated in top-down control of emotion and decision-making and is thought to modulate subcortical structures such as the central and basolateral amygdala [29, 41, 42] and the ventral and dorsal striatum [12, 43–45] that are implicated in valence processing and defensive behaviors including active avoidance [39, 46, 47]. Indeed, greater synchrony between mPFC and both the amygdala and striatum predicts effective avoidance learning [30]. Amygdala-striatal circuitry is also thought to be important for avoidance learning [35], although some studies indicate that the amygdala may no longer be required for avoidance expression after extensive training, suggesting that frontostriatal mechanisms of habitual avoidance may dominate after avoidance is well learned [29]. However, the majority of work on frontostriatal circuitry in instrumental learning has used *positive reinforcement* [48–51], whereas active avoidance learning can be a form of *negative reinforcement*. Whether the frontostriatal circuit mechanisms underlying positive reinforcement are also relevant to avoidance remains an open question of critical importance to understanding avoidance mechanisms [52].

In contrast to active avoidance, passive avoidance involves withholding a behavioral response in order to prevent harm. Passive avoidance has traditionally been conceptualized as a readout of fear memory [53–56], and work in rodents and non-human primates have implicated the amygdala and extended amygdala in passive defensive responding [57–59]. However, little is known mechanistically about passive avoidance behavior at the circuit level, likely because it is difficult to identify neural correlates in the absence of a behavior. In addition, few studies have directly compared active and passive avoidance behavior to identify similarities and differences in neural mechanisms (though see [45, 60] for paradigms that facilitate this comparison).

## Maladaptive avoidance

While much is known about the neural circuitry underlying *adaptive* avoidance (i.e., avoidance of a truly noxious stimulus), anxiety disorders, OCD, and PTSD are typically characterized by *maladaptive* avoidance. We define maladaptive avoidance as avoidance of a relatively safe stimulus and/or resulting in negative consequences for the individual, such as loss of reward. To model avoidance of a relatively safe stimulus, paradigms typically examine either extinction-resistant avoidance, or generalization of avoidance to a cue perceptually similar to a conditioned stimulus. To model negative consequences from avoidance, paradigms often introduce competing rewards that the individual must forgo to perform the avoidance behavior. Common and promising examples of paradigms that model maladaptive avoidance are reviewed below and summarized in Table 1.

**Table 1:** Selected examples of common and/or promising maladaptive avoidance paradigms.

Selected Paradigms	What makes it maladaptive avoidance		Likely neurobehavioral process(es) involved			Examples		Brain regions implicated
	Avoidance in relative safety?	Avoidance has a negative consequence?	Heightened threat appraisal	Habitual avoidance	Trait avoidance tendency	Animal models	Human	
Extinction-resistant avoidance	X		X	X		Bravo-Rivera et al 2015	Vervliet et al 2015	mPFC, ventral striatum, BLA
Extinction-resistant avoidance with overtraining	X			X		Martinez - Rivera et al 2020	Gillan et al 2014 and 2015	Frontostriatal circuitry, caudate
Extinction-resistant avoidance with response prevention	X			X		Rodriguez - Romaguera et al 2016	Vervliet & Indekeu 2015	Lateral OFC
Avoidance generalization	X	X	X		X	–	van Meurs et al. 2014; Dymond et al 2014	–
Platform-mediated avoidance test		X			X	Bravo-Rivera et al 2021	–	BLA, mPFC
Semi-naturalistic closed economy	X	X				Fanselow et al 1988; Kim et al. 2014	–	BLA
Threat discounting paradigm	X	X			X (also reward sensitivity)	–	Pittig & Scherbaum 2020; Aupperle et al 2015	Anterior insula, mPFC

Abbreviations: *mPFC* Medial prefrontal cortex; *BLA* Basolateral amygdala; *OFC* Orbitofrontal cortex.

## Extinction-resistant avoidance

Extinction-resistant avoidance is defined as avoidance behavior that persists even after Pavlovian extinction training in which the CS (cue) is repeatedly presented in the absence of the US (aversive stimulus), and is maladaptive because the CS no longer predicts threat. Extinction-resistant avoidance has clear clinical relevance because continued avoidance following exposure therapy (which is thought to work through extinction) may put patients at higher risk for relapse [61, 62]. However, extinction-resistant avoidance could be due to more than one underlying neurobehavioral process. Extinction-resistant avoidance may be due to heightened threat appraisal, particularly if both fear *and* avoidance persist following extinction training. Such incomplete extinction of both fear and avoidance is more common when avoidance behavior is not prevented during extinction training. In this case, animals continue to avoid during extinction training, thereby receiving fewer opportunities to extinguish the CS-US association and resulting in persistent fear [63]. Alternatively, despite the often strong coupling between fear and avoidance, extinction-resistant avoidance behavior can occur even when extinction training has successfully reduced conditioned fear responding [63, 64]. In these cases, avoidance may have become a habit and thus governed by different neural circuitry.

Animal studies of extinction-resistant avoidance behavior have largely built on active avoidance paradigms. After avoidance learning, animals undergo extinction training in which the CS is presented in the absence of the US. Although most animals learn that there is no longer a need to avoid the CS, there are large individual differences in avoidance following extinction training, with a substantial fraction of animals continuing to persistently avoid [63, 64]. However, it is hard to distinguish whether this extinction-resistant avoidance is simply a product of insufficient fear extinction without measuring both fear and avoidance. Human studies of extinction-resistant avoidance have more consistently measured both fear and avoidance, allowing stronger conclusions to be drawn about the neurobehavioral process underlying the observed avoidance behavior. For example, greater avoidance behavior has been observed in OCD patients than in healthy controls, in the absence of fear-related differences indexed by skin conductance [65]. Furthermore, greater avoidance in OCD was only seen after a large number of avoidance trials, suggesting a habitual component to the avoidance. In contrast, extinction-resistant avoidance behavior has been observed in the context of incomplete extinction of fear, which suggests that this persistent avoidance behavior may be driven by continued threat appraisal [36]. Further supporting the notion that a threat-related process drives avoidance was the relationship between trait anxiety and extinction-resistance avoidance, as well as the relatively small number of avoidance training trials in this study [36].

Another approach to disentangling the relative contribution of threat appraisal and habit in extinction-resistant avoidance is using response prevention (i.e., removing the opportunity to avoid during extinction training) to ensure that fear responses are completely extinguished. Rodent studies have directly compared neural correlates of persistent avoidance following extinction training with and without response prevention. Extinction-resistant avoidance was correlated with increased activity in the prelimbic cortex and ventral striatum regardless of response prevention, but when response prevention was absent, extinction-resistant avoidance was also correlated with impaired recruitment of regions associated with fear extinction (i.e., increased basal amygdala and decreased infralimbic cortex activity) [63]. These results suggest that without response prevention, animals may not have sufficient opportunity to extinguish fear, and this heightened threat appraisal may be driving persistent avoidance. In contrast, persistent avoidance following extinction *with* response prevention may be due to habitual avoidance (though spontaneous recovery of fear or lack of cost for continuing to perform the avoidance response may also contribute). Habitual avoidance has been particularly implicated in OCD, and clinical treatment of OCD typically involves exposure to feared stimuli (e.g., dirty objects) combined with response prevention of compulsive avoidance behavior (e.g., hand-washing). A recent study showed that overtraining on avoidance behavior biased rats toward habitual avoidance following extinction training with response prevention, which was associated with increased activity in frontostriatal regions [66]. This paradigm is a promising model of habitual avoidance that may be present in OCD, and presents an opportunity for further investigation into circuit mechanisms underlying extinction-resistant avoidance. Furthermore, at least one inactivation study in rodents suggests that extinction-resistant avoidance following response prevention depends on the lateral orbitofrontal cortex [67], which has been implicated in both habit [68] and OCD [69]. Functional neuroimaging of adults with OCD has also implicated the caudate nucleus in persistent habitual avoidance in this population [70].

## Avoidance generalization

Generalization is a phenomenon in which individuals apply learning from past experiences to similar related situations or stimuli [71]. Generalization can be adaptive by allowing us to navigate complex risks based on limited experience. However, over-generalization of avoidance behavior is maladaptive, as it leads to avoidance of related but safe situations. Avoidance over-generalization is a core feature of anxiety and related disorders [13]. For example, someone with PTSD as a result of a car accident may not only avoid the intersection where the crash occurred, but may generalize to avoiding driving altogether. Avoidance generalization could be driven by the underlying processes of heightened threat appraisal, if the avoidance generalization is proportional to the generalization of fear, or by trait avoidance tendency.

Although few rodent studies have examined *avoidance* generalization, many have focused on *fear* generalization. These studies have shown that tonic activity of central amygdala neurons correlates with the degree of fear generalization [72], and increased synchrony between basolateral amygdala and medial prefrontal neurons occurs in animals demonstrating less fear generalization [73]. However, little is known about whether these same circuits regulate generalization of avoidance responses. In contrast to the limited rodent models of avoidance generalization, there has been a surge of interest in human avoidance generalization paradigms (e.g., [74–76]). For example, in the virtual farmer game [77], participants first undergo Pavlovian conditioning to associate a shape (CS) with a shock. Next, participants must move a farmer icon between images of a shed and crops, aiming to arrive at the crops before virtual birds interfere. They are given the option of a short path, which results in shock when the CS is present, or a long path, which interferes with the goal of the game but is always safe from shock. Avoidance generalization is measured by the extent to which participants select the long path when generalization stimuli (with some perceptual similarity to the CS but never paired with shock) are present.

As with extinction-resistant avoidance, experimentally measuring both fear and avoidance in the same tasks will help distinguish the underlying neurobehavioral processes driving avoidance generalization. In the virtual farmer paradigm, individuals who exhibited greater fear generalization (measured by skin conductance) demonstrated greater avoidance generalization (measured by selecting the long path), suggesting that heightened threat appraisal may be a driver of avoidance generalization in this paradigm [77]. This paradigm could also be used to identify individual differences in trait avoidance tendency, for example, if individuals avoid a generalization stimulus in the absence of significant fear responding. Greater avoidance generalization in the virtual farmer game was associated with individual differences in avoidant coping styles such as distraction or suppression [77], consistent with the notion that trait avoidance tendency may play a role in avoidance generalization for some individuals. Neural mechanisms of avoidance generalization are not well understood relative to other paradigms (Table 1). Extending animal models of fear generalization to avoidance behavior would help delineate neural mechanisms, and is an important future direction.

## Competing rewards

Maladaptive avoidance behavior often results in negative consequences such as loss of opportunity for positive outcomes. Thus, a common strategy to study maladaptive avoidance behavior is to pit avoidance against a competing reward, such that avoidance results in both a decreased risk of harm *and* a decreased potential for reward. This approach parallels clinical situations such as in social anxiety, where avoidance of social interaction results in loss of both potential negative outcomes (e.g., being rejected) and potential positive outcomes (e.g., making friends). Avoidance in the face of competing reward could be driven by either acquired habitual avoidance, or an innate imbalance between trait avoidance tendency and reward sensitivity. If behavior is habitual, it is by definition not goal-directed and will persist regardless of the reward accrued. In addition, individuals may differ in the extent to which they are either predisposed to avoid and/or sensitive to the potential for reward, either of which would result in greater avoidance decisions when competing rewards are present [78].

One example of how competing rewards can be implemented in animal models is the platform-mediated avoidance test [28], in which animals can press a lever for a food reward as long as they are on the shock floor. When they step onto the platform to avoid the shock, however, they are no longer able to reach the lever and therefore forgo this reward. This task has been further modified to maximize competition between avoidance and reward by presenting the lever for reward only during presentation of the tone that signals impending shock. There were individual differences in avoidance tendency such that rats that preferred avoidance had increased amygdala activity compared to rats that preferred reward approach, which had decreased prefrontal activity [79]. The “risk-reward interaction” task [60] also adds an element of reward into a traditional shuttle box style apparatus, such that each chamber contains a shock floor and a reward port. This task has been used to measure neural signals related to active avoidance and reward-seeking behaviors on separate trials but could also be adapted to make reward only accessible by crossing a shock floor. This would allow researchers to measure how much an animal is willing to forgo reward in order to avoid, and examine if different neural mechanisms are involved when avoidance is paired with competing rewards. Variations of the risk-reward interaction task could also be used to assess how decisions to forgo rewards relate to the acquisition of avoidance habits, and whether different neural mechanisms are involved in this decision once avoidance has become habitual. In addition, although these paradigms have thus far been used with the fixed intensity of threats and rewards, varying the intensity could better probe individual thresholds for maladaptive avoidance behavior. A complementary approach to these acute assays is the semi-naturalistic “closed economy” paradigm in which animals live for several weeks in the apparatus and must forage for food in a location that also delivers random electric shocks, allowing for ethologically relevant probing of avoidance behaviors as a reward and/or shock schedules are varied [80–82].

Human studies have begun investigating the impact of varying threat and reward intensities using computer games that provide participants the opportunity to earn rewards (e.g., money) by facing negative consequences, or avoid these consequences without receiving any reward [78, 83]. While related work has investigated *loss* of money as a negative consequence [84, 85], we focus here on paradigms involving the *addition* of an aversive outcome, such as viewing threatening images or receiving the shock. In particular, recent work examined avoidance decision-making under varying combinations of shock probability and reward magnitude [83]. High anxiety individuals made more avoidance decisions compared to low anxiety individuals as reward magnitude increased, suggesting that avoidance decisions in anxious individuals may be driven by low reward sensitivity. Although the neural mechanisms underlying this paradigm have not been examined, functional neuroimaging studies of similar paradigms have implicated the anterior insula and medial prefrontal cortex [16, 86]. In non-human primates, amygdala lesions increase avoidance in the presence of competing rewards [87], while stimulation of the anterior insula and ventral striatum increases this avoidance behavior [88].

A related type of paradigm is an approach-avoidance conflict task, which presents an animal with conflicting appetitive and aversive cues that must be simultaneously weighed. In contrast to the examples above, which use *learned* avoidance behavior, approach-avoidance conflict tasks have traditionally examined *innate* avoidance behavior. The most common approach-avoidance conflict assay is the elevated plus maze, which is a platform consisting of two “open” exposed arms and two “closed” arms with tall walls that animals freely explore [89, 90]. This task capitalizes on rodents’ conflicting innate aversion to open brightly lit spaces (for risk of predation) and innate drive to explore novel environments. There are many related tasks to study conflicting costs and benefits. For example, the T-maze measures how willing an animal is to avoid an innately aversive stimulus, such as bright light, at the cost of losing a reward, such as food [91]. Semi-natural foraging environments that employ food rewards and robotic predators have likewise been used to study approach-avoidance conflict behaviors [58]. In addition, human and non-human primate studies have used a joystick to capitalize on the instinct to pull desired stimuli closer and push aversive stimuli away [92]. Due to the innate nature of the avoidance behavior in approach-avoidance conflict tasks, greater maladaptive avoidance in these tasks can be considered a measure of trait avoidance tendency.

Approach-avoidance conflict tasks require the mPFC: ventromedial aspects that project to the basolateral amygdala control open arm exploration in the elevated plus maze [47], whereas dorsomedial aspects that project to the dorsal striatum control cost-benefit decision-making in the T-maze task as well as innate avoidance in the elevated plus maze [91, 93]. This suggests an important role of frontostriatal projections in trait avoidance tendency. There is also some encouraging evidence that these tasks have relevance for human anxiety. Human analogues of approach-avoidance conflict tasks have used movement tracking to measure innate avoidance versus exploration behavior in real or virtual settings [94–97]. These studies have generally found greater innate avoidance behavior in those with higher anxiety, and decreased avoidance following anxiolytic medication, validating the potential utility of this approach for understanding anxiety disorders and trait-like individual differences.

## Gaps in knowledge

Moving forward, it will be important to address four key areas: differentiating neurobehavioral alterations, translating insights across species, testing paradigms in clinical populations, and improving diagnosis and treatment (Table 2).

**Table 2:** Four proposed areas for ongoing development, and corresponding key unanswered questions.

Areas for development	Key questions
Differentiating neurobehavioral alterations	1. Are the three neurobehavioral processes we have proposed independent routes to generating maladaptive avoidance behavior? 2. Which circuit dysfunctions underlie these different types of maladaptive avoidance?
Translating insights across species	1. Do the same neural substrates support instructed avoidance behavior (observable in humans only) and learned avoidance behavior?2. How do neural mechanisms in animal models compare with neural mechanisms in humans when paradigms are matched on behavior and/or autonomic physiology?
Testing paradigms in clinical populations	1. How do clinically anxious individuals differ from healthy controls in dynamics of learning, expression, and extinction of maladaptive avoidance behavior? 2. How does avoidance behavior and its neural substrates differ across anxiety disorders and related diagnoses?
Improving diagnosis and treatment	1. Which maladaptive avoidance paradigms reliably quantify individual differences in avoidance? 2. Which interventions most effectively decrease maladaptive avoidance behavior measured in such laboratory paradigms? 3. Can identifying the specific neurobehavioral process underlying avoidance in a patient help indicate which treatment will be effective for them?

### Differentiating neurobehavioral alterations

**Fig. 1:**
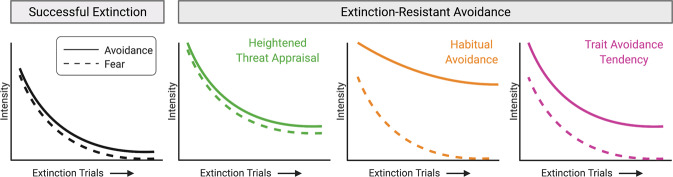
Illustration of how the relationship between fear and avoidance could differentiate underlying neurobehavioral processes. One example of a paradigm that could be modified to better differentiate underlying neurobehavioral processes is extinction-resistant avoidance. Successful extinction results in attenuation of both fear and avoidance responding (dashed and solid lines, respectively). In contrast, extinction-resistant avoidance involves continued avoidance behavior following extinction training. We hypothesize that the underlying neurobehavioral process driving the persistent avoidance—e.g., heightened threat appraisal (green), habitual avoidance (orange), or trait avoidance tendency (magenta) -- could be better differentiated by examining both fear and avoidance responding over time.

A major gap in the field is the lack of translational paradigms that can differentiate which neurobehavioral alterations underlie a particular individual’s maladaptive avoidance behavior. An important next step will therefore be to develop tasks that better differentiate the processes and circuitry underlying maladaptive avoidance. Existing paradigms could be modified to determine the extent to which fear and avoidance are correlated by including quantifiable proxies for fear (e.g., autonomic measures such as heart rate or skin conductance) and investigating how closely they track with avoidance behavior over time. A close coupling between fear and avoidance would suggest a greater contribution of threat appraisal to the avoidance behavior, whereas lower coupling would imply that habitual avoidance and/or avoidance tendency are involved (Fig. 1). In addition, examining the temporal dynamics of avoidance could help differentiate underlying processes. For example, because habits are less sensitive to action-outcome relationships, avoidance behavior driven by habit would be less likely to diminish during extinction training (Fig. 1).

In animal studies, differentiating between these specific underlying processes will require longitudinal tracking of both behavior and neural activity at a resolution that has not yet been brought to the field. Greater specificity in differentiating underlying processes will also require measuring defensive behaviors beyond avoidance (e.g., freezing), and recording neural activity not only during avoidance expression but also during avoidance learning and extinction. Combining these behavioral measurements with cell-type-specific and neural projection-specific circuit dissection techniques, such as optogenetics and in vivo calcium imaging, will be critical for elucidating the real-time circuit dynamics correlated with each of these behaviors.

### Translating insights across species

Animal models can provide greater spatial and temporal precision in identifying neural mechanisms, while human research can assess internal experiences (e.g., feeling anxious), and provide verbal instruction on available avoidance behaviors. Such instructed avoidance paradigms may allow for better separation of trait avoidance tendency and habit because instructed avoidance behavior removes the possibility that repeated practice of avoidance has led to habitual responding. However, while these unique insights are important, it is also important to align paradigms across human and animal models to better link the unique insights from each species. This should include alignment on the precise use of terminology, such as “relief” (a clinically-relevant subjective experience) versus “shock omission” (an objectively defined event). Human and animal paradigms will have better alignment when they involve common behavioral outputs and physiological measures during avoidance tasks. Additionally, quickly-developing technology such as virtual reality platforms [98] and smartphone-based passive sampling [99] offer a unique ability to align tasks across species by re-creating for humans the freely moving laboratory tasks from rodent work. Computational models that can generate hypotheses independent of the species being studied are another important approach to facilitate translation [100]. Finally, while the majority of research has focused on rodent and human models of avoidance behavior, more work is needed to translate across non-human primate and human studies, given the advantage of greater functional homology of the brain relative to rodent models [101, 102].

### Testing paradigms in clinical populations

Another crucial gap is understanding how avoidance behavior as defined in laboratory paradigms applies in clinical populations. It remains unclear whether the mechanisms uncovered from studies of adaptive avoidance are the same as those that go awry in clinical conditions that are defined by maladaptive avoidance. The majority of paradigms described above have not been tested in individuals with clinical levels of anxiety and avoidance; doing so would allow for a more nuanced characterization of maladaptive avoidance behavior within and across disorders. For example, the distinction between active and passive avoidance strategies is commonly made in animal models, yet rarely made in describing clinical phenomena, highlighting a missing translational link. Moreover, it is not known whether mechanisms underlying external avoidance (e.g., of electric shock) are the same as those underlying experiential avoidance (e.g., of unpleasant thoughts or emotions) common in anxiety and related disorders. Despite large interest in trans-diagnostic features of psychopathology, it is still unclear whether avoidance involves the same neural mechanisms across disorders. For example, does avoidance of social situations in social anxiety disorder involve the same mechanisms as avoidance of trauma reminders in PTSD? Understanding how specific neurobehavioral processes map onto existing DSM diagnoses or trans-diagnostic features (such as worry or intolerance of uncertainty) will be an important step to bridge current clinical practice and a neuroscience-based understanding of maladaptive avoidance behavior. Finally, it will be important for the field to develop paradigms that mimic the probabilistic nature of safety in the real world, in addition to existing experimental laboratory paradigms that can more objectively define safety.

### Improving diagnosis and treatment

Assuming that distinct neurobehavioral processes reflect different root causes of maladaptive avoidance behavior, an open question is whether they could someday provide a more precise and useful classification system than a current clinical practice using DSM symptom-based classification. This goal would require reliable and valid measurement of avoidance behavior at the level of an individual, with known population norms and/or clinical cut points. While some studies have begun to examine construct validity [36, 53], psychometric properties of avoidance paradigms have largely not been established (for a notable exception, see [103]). Psychometrically sound tasks will be essential for translation from research to the clinical application [104], with test-retest reliability of particular importance for paradigms assessing constructs that should be stable over time, such as trait avoidance tendency. Using a classification system based on reliable and valid measurement of underlying neurobehavioral alterations, we may be able to better tailor interventions to target specific avoidance problems unique to each patient. Although the current gold-standard treatment approach for anxiety and related disorders is exposure therapy, this treatment is not successful for many patients [105]. Exposure therapy is thought to work through fear extinction [106]; this extinction-based view of exposure therapy suggests that exposure therapy will be most effective in addressing avoidance behaviors driven by heightened threat appraisal. However, it is unclear whether exposure therapy can also effectively modify avoidance behavior that has become decoupled from fear, as with trait avoidance tendency or habitual avoidance, or whether maladaptive avoidance behavior driven by these processes could be better addressed by operant reinforcement principles rather than Pavlovian and inhibitory learning processes. For example, one hypothesis is that interventions such as acceptance and commitment therapy (ACT) that focus on approach towards values-based behaviors [62, 107] may be more effective treatments for trait avoidance. While one study has found that avoidance is associated with more success in CBT than ACT [9], the neurobehavioral process underlying this avoidance is unclear. Ultimately, just as insights from the laboratory paradigm of fear extinction have improved exposure therapy [106], laboratory paradigms that can measure and manipulate maladaptive avoidance have the potential to identify and optimize new treatment approaches to more directly target additional root causes of maladaptive avoidance behavior.

## Conclusion

Avoidance is a central and trans-diagnostic mechanism that leads to impairment across anxiety pathology, including OCD and PTSD. Reducing maladaptive avoidance is an important treatment goal and a necessary part of evidence-based interventions such as exposure therapy. We propose that maladaptive avoidance may be caused by alterations in three distinct neurobehavioral processes: (i) threat appraisal, (ii) habitual avoidance, and (iii) trait avoidance tendency. Existing paradigms to measure maladaptive avoidance have examined extinction-resistant avoidance, avoidance generalization, and avoidance in the face of competing rewards. An important next step for the field will be to adapt these paradigms to more precisely differentiate which neurobehavioral alterations underlie a particular individual’s maladaptive avoidance behavior and use these paradigms to understand how specific neurobehavioral processes map onto clinical diagnoses. Ultimately, this work has the potential to lead to new interventions to more effectively treat maladaptive avoidance behavior in anxiety and related disorders.
